# Development of rosuvastatin flexible lipid-based nanoparticles: promising nanocarriers for improving intestinal cells cytotoxicity

**DOI:** 10.1186/s40360-020-0393-8

**Published:** 2020-02-21

**Authors:** Tarek A. Ahmed

**Affiliations:** 1 Department of Pharmaceutics, Faculty of Pharmacy, King Abdulaziz University, Jeddah, 21589 Kingdom of Saudi Arabia; 2 Department of Pharmaceutics and Industrial Pharmacy, Faculty of Pharmacy, Al-Azhar University, Cairo, Egypt

**Keywords:** Rosuvastatin, Optimization, Chitosomes, Liposomes, cell viability, Cytotoxicity

## Abstract

**Background:**

Rosuvastatin (RSV) is a poorly water-soluble drug that has an absolute oral bioavailability of only 20%. The aim of this work was to prepare a positively charged chitosan coated flexible lipid-based vesicles (chitosomes) and compare their characteristics to the corresponding negatively charged flexible liposomal nanoparticles (NPs) in order to develop new RSV nanocarrier systems.

**Methods:**

Three formulation factors affecting the development of chitosomes nano-formulation were optimized for their effects on the particles size, entrapment efficiency (EE) and zeta potential. The optimized flexible chitosomes and their corresponding liposomal NPs were characterized for morphology, in vitro release, flexibility and intestinal cell viability. The half maximum inhibitory concentrations (IC50) for both formulations were calculated.

**Results:**

The drug to lipid molar ratio, edge activator percent and the chitosan concentration were significantly affecting the characteristics of NPs. The optimized chitosomes nano-formulation exhibited larger size, higher EE and greater zeta potential value when compared to the corresponding liposomal NPs. Both formulations showed a spherical shape nanostructure with a marked outer shell for the chitosomes nano-formulation. Chitosomes illustrated an extended drug release profile when compared with the corresponding liposomal NPs and the prepared drug suspension. Flexibility of both vesicles was confirmed with superiority of liposomal NPs over chitosomes. RSV loaded chitosomes nano-formulation exhibited lower IC50 values and higher therapeutic window while liposomal NPs were compatible with the intestinal cells.

**Conclusions:**

RSV loaded chitosomes nano-formulation could be considered as a promising nanocarrier system with a marked cytotoxic activity while, RSV loaded liposomal NPs are suitable nanocarrier to improve RSV activity in treatment of cardiovascular disorders.

## Background

Rosuvastatin (RSV), a member of statins, is used to prevent cardiovascular disorders by decreasing the low-density lipoprotein (LDL) cholesterol. It is the most effective hypolipidemic agent of the statins group and has been assigned the name super-statin [4, 6]. Similar to other statins, the mechanism of action of RSV is attributed to competitive inhibition of the enzyme 3-hydroxy-3-methyl-glutaryl-CoA (HMG-CoA) reductase [30]. RSV has a low water solubility and exhibits a limited solubility in the gastrointestinal fluids [11]. The drug is subjected to extensive first pass metabolism after oral administration. Accordingly, the oral bioavailability of RSV is approximately 20%. The maximum RSV plasma concentration is reached in about 3 to 5 h. The drug is 88% bound to plasma protein mainly to serum albumin. It is probable that about 25% of the orally administered dose is absorbed [27, 45]. RSV is mainly metabolized by the liver CYP2C9. It is 90% excreted in feces and the drug elimination half-life is nearly 19 h.

Different pharmaceutical formulation strategies have been used to enhance the bioavailability of drugs that are suffering from poor aqueous solubility. These include size reduction, the use of cosolvents and surfactants, solid dispersion and inclusion complexation techniques, salt formation and prodrug approaches. Moreover, formation of colloidal drug delivery systems such as solid lipid nanoparticles (NPs), polymeric based NPs, lipid based NPs, microemulsion formation and self-microemulsifying drug delivery systems has been reported [26, 31]. Flexible liposomes have been assigned the name “Transfersomes” and these NPs constitute a class of the lipid based NPs which consist of phospholipid(s) and a single chain surfactant [12]. The presence of surfactant (edge activator) promotes flexibility of these NPs by reducing the rigidity of the phospholipid bilayer and so render these nanocarriers ultra-deformable vesicles [1]. Due to their successful delivery of a wide variety of pharmacologically active agents, these ultra-deformable NPs have attracted too much research interest [1, 9, 12, 13, 18, 20, 22, 25, 39].

Chitosan is a linear polysaccharide polymer that is obtained from chitin shells of shrimp and other crustaceans after treatment with an alkaline substance such as sodium hydroxide. It is consists of a randomly distributed β-(1 → 4)-linked D-glucosamine unit and N-acetyl-D-glucosamine unit. The former unit is the deacetylated part while the latter is the acetylated portion in the chitosan structure. It is the only naturally occurring alkaline polysaccharide polymer with good biocompatibility and biodegradability [43]. Different chitosan based NPs have been developed and reported to be an effective drug delivery systems due to their efficient drug encapsulation, protection against enzymatic degradation, enhanced drug permeation and controlled release action [2]. Moreover, NPs coated with chitosan have been reported in the literature to enhance stability and drug loading, controlling the drug release and maximizing the efficacy. Chitosan-coated poly (lactic-co-glycolic acid) NPs, magnetic NPs coated with chitosan, chitosan-coated cobalt ferrite NPs, chitosan-coated solid lipid NPs and chitosan-coated liposomes are common examples [5, 8, 14, 17, 21, 24, 42].

In this study, a promising RSV flexible lipid-based nanocarriers, liposomal NPs and chitosomes nano-formulation, were successfully prepared and characterized for vesicles size, zeta potential, entrapment efficiency, morphology, flexibility, in vitro drug release, cell viability and cytotoxicity. The half maximum inhibitory (IC50) concentrations for both nanocarrier systems were calculated to investigate their therapeutic window. The stability, pharmacokinetic and pharmacodynamic activity of these drug loaded flexible lipid-based NPs will be investigated, in our upcoming work, after loading into the suitable drug delivery system.

## Materials and methods

Rosuvastatin (RSV) was obtained as a gift from the Saudi Arabian Japanese Pharmaceuticals Co. Ltd. (SAJA) (Jeddah, KSA). Dicetyl phosphate (DCP) and methanol were purchased from Fisher Scientific (Pittsburgh, PA, USA). Tween 80, low molecular weight chitosan and glacial acetic acid were procured from Sigma-Aldrich (St. Louis, MI, USA). L-α phosphatidylcholine (95%) (soy), molecular weight = 775.037 (average based on fatty acid distribution in product) was purchased from Avanti® polar lipids, INC. (Alabaster, Alabama, USA). All other materials used were of analytical grade.

### Box-Behnken experimental design

StatGraphics Centurion XV version 15.2.05 software, StatPoint Technologies, Inc., (Warrenton, VA, USA) was employed to study the effect of three independent factors affecting the development of RSV chitosan-coated flexible lipid-based nanocarrier (chitosomes nano-formulation). The drug to phospholipid molar ratio (X_1_), the surfactant (edge activator) concentration (X_2_) and the chitosan coating solution concentration (X_3_) were studied for their effect on vesicle size (Y_1_), entrapment efficiency (Y_2_) and zeta potential (Y_3_). Fifteen formulations were proposed, and their composition is illustrated in Table 1. The studied independent factors namely; X_1_, X_2_ and X_3_ were used at 1:1–1:4 drug to phospholipid molar ratios, 0.01–0.04% (w/v) based on the total formulation volume and 0.2–0.6% (w/v), respectively. The goal was to minimize Y_1_ and maximize both Y_2_ and Y_3_.

**Table 1:** Composition of rosuvastatin chitosomes nanoparticles formulations and the obtained values for the studied responses

Run	X_1_ (MR)	X_2_ (%)	X_3_ (%)	Y_1_		Y_2_ (%)	Y_3_ (mV)
				Size (nm)	PDI		
1	1.0	0.01	0.4	239.48 ± 5.33	0.344 ± 0.065	90.88 ± 1.65	12.66 ± 1.58
2	4.0	0.04	0.4	225.27 ± 6.59	0.425 ± 0.095	86.94 ± 4.94	18.10 ± 3.38
3	2.5	0.04	0.2	112.19 ± 2.90	0.288 ± 0.009	85.14 ± 5.04	− 1.83 ± 0.55
4	2.5	0.01	0.6	554.33 ± 11.06	0.573 ± 0.045	94.59 ± 1.62	26.74 ± 0.26
5	2.5	0.04	0.6	507.40 ± 18.14	0.581 ± 0.051	91.97 ± 4.89	26.07 ± 1.90
6	4.0	0.01	0.4	295.40 ± 5.11	0.258 ± 0.023	88.75 ± 3.99	10.70 ± 0.16
7	4.0	0.025	0.6	547.33 ± 20.09	0.511 ± 0.024	92.79 ± 1.76	28.87 ± 1.39
8	2.5	0.01	0.2	163.13 ± 3.29	0.391 ± 0.053	87.94 ± 2.04	− 8.14 ± 0.51
9	1.0	0.025	0.6	535.33 ± 43.12	0.543 ± 0.053	93.22 ± 1.24	27.23 ± 0.31
10	1.0	0.025	0.2	132.77 ± 4.46	0.439 ± 0.061	87.61 ± 2.07	−10.07 ± 1.26
11	1.0	0.04	0.4	219.53 ± 6.36	0.354 ± 0.071	90.63 ± 2.15	13.15 ± 0.75
12	4.0	0.025	0.2	132.97 ± 2.11	0.318 ± 0.022	84.78 ± 3.09	−6.92 ± 0.16
13	2.5	0.025	0.4	255.83 ± 8.37	0.394 ± 0.128	85.44 ± 2.21	11.26 ± 2.04
14	2.5	0.025	0.4	252.30 ± 3.84	0.318 ± 0.088	86.38 ± 3.03	11.83 ± 2.67
15	2.5	0.025	0.4	259.77 ± 3.69	0.382 ± 0.043	85.15 ± 4.07	14.87 ± 1.89

**Abbreviations:** *X*_*1*_ Drug to phospholipid, *X*_*2*_ Surfactant concentration, *X*_*3*_ Coating solution concentration, *Y*_*1*_ Particle size (nm,*Y*_*2*_ Entrapment efficiency (%), *Y*_*3*_ Zeta potential (mV), *MR* Molar ratio, *PDI* Poly dispersity index

### Preparation of RSV flexible-lipid based NPs

Lipid film hydration technique was used to prepare the NPs formulations according to the method previously reported in our previously published work but with little modifications [1]. Briefly, 100 mg of RSV and the calculated amount of phospholipid, edge activator (tween 80) and DCP (15% w/w of the total lipid) were dispersed in 100 mL methanol in a Buchi rotavapor evaporating flask. The mixture was subjected to sonication using Ultrawave Ltd., CF3 2EY water bath sonicator (Cardif, UK) until homogenous dispersion was obtained. The organic solvent, methanol, was evaporated at a temperature of 50 °C under reduced pressure and continuous rotation using Buchi Rotavapor R-200, Buchi labortechink AG, CH-9230 (Flawi, Switzerland). The obtained lipid film deposited inside the flask wall was kept for 24 h in a vacuum oven of Thermo Fisher Scientific, model 6505 (Oakwood Village, OH, USA) to ensure complete evaporation of methanol. The hydration medium, 50 mL of phosphate buffer pH 7, was added to the flask that was kept shaking in the rotavapor for 30 min at 50 °C. The obtained flexible liposomal dispersion was subjected to probe sonication using Sonics Vibra cell, VCX 750; Sonics & Materials, Inc. (Newtown, CT, USA) for 10 min at an amplitude of 60%.

For the coating step, three chitosan solutions (0.4, 0.8 and 1.2% w/v) were prepared by dissolving the calculated amount of chitosan in 0.5% v/v acetic acid solution. According to the formulation composition, 50 mL of the specified chitosan solution was added dropwise to an equal volume of the prepared flexible liposomal dispersion over a magnetic stirrer at 1200 rpm for 2 h at room temperature. The prepared chitosan-coated flexible liposomal NPs (chitosomes nano-formulation) of 0.2, 0.4 or 0.6% w/v chitosan were left over night in the refrigerator for complete curing of the NPs.

A specified volume of the obtained flexible liposomal NPs was separated and characterized, as will described in the following section, to compare their features with the corresponding chitosomes nano-formulation.

### Characterization of the flexible lipid-based nanoparticles

#### Particle size distribution and zeta potential

The particle size, polydispersity index (PDI) and zeta potential for the prepared fifteen NPs formulations were determined (*n* = 3) using Malvern Zetasizer Nano ZSP, Malvern Panalytical Ltd. (Malvern, United Kingdom). Dynamic light scattering with non-invasive backscatter optics was the technique utilized in the measurement.

#### Entrapment efficiency (EE)

The obtained RSV loaded flexible lipid-based nanocarriers (liposomal NPs and chitosomes nano-formulation) were centrifuged at 20000 rpm for 1 h at 4 °C using 3 K30 Sigma Laboratory centrifuge (Ostrode, Germany) to separate the free unentrapped drug. The supernatant was filtered through 0.2 μm Millipore filter and the drug concentration was estimated spectrophotometrically at 242 nm using 6705 UV/Vis spectrophotometer, Jenway (Stone, UK). The EE (%) for each formulation was calculated indirectly using the following equation.

$$ EE\ \left(\%\right)=\frac{Total\ amount\ of\ drug\ used- Calculated\ amount\ of\ free\ drug\kern0.5em }{Total\ amount\ of\ drug\ used}\times 100 $$

Accuracy of the spectrophotometric method and its freedom from any possible interference by the formulation components were verified. Recovery testing for RSV concentration in different drug solutions containing the studied excipients was verified.

### Box-Behnken experimental design statistical analysis

Data obtained for particle size, EE and zeta potential for the prepared flexible chitosomes nano-formulation were statistically analyzed to identify the main, interaction and quadratic effects significantly affecting each response. The effect was considered significant at *p*-value less than 0.05. An optimized formulation that achieve the study goal was proposed.

### Preparation and characterization of the optimized formulation

The proposed optimized NPs formulation was prepared and characterized for size, PDI, EE and zeta potential as described above. The observed values were compared to the predicted ones and the residual was calculated.

### Morphological study

Few drops of the optimized flexible-chitosomes nano-formulation was mounted on a carbon coated grid and left for 5 min to allow better adsorption of the NPs on the carbon film. Excess liquid was removed by a filter paper. Few drops of 1% phosphotungstic acid was added and the sample was examined under the transmission electron microscopy (TEM), Model JEM-1230, JOEL (Tokyo, Japan). Morphology of the corresponding liposomal NPs formulation was also studied and compared to the chitosomes nano-formulation.

### Fourier transforms infrared (FT-IR)

The FT-IR spectra of pure RSV, phospholipid and chitosan samples were studied using a Nicolet Is10 of Thermo Scientific, Inc., (Waltham, MA). The spectra of the prepared liposomal NPs and chitosomes nano-formulation were also investigated to identify any possible changes in the drug physicochemical characteristics following development of the flexible lipid-based NPs formulations. The FT-IR spectra of all the studied samples were recorded in the range of 4000–400 Cm^− 1^.

### In vitro release study

The in vitro release of RSV from the prepared liposomal NPs, chitosomes nano-formulation and pure drug suspension was studied as previously reported [5]. A quantity of each preparation containing 9 mg of drug was placed in a firmly sealed dialysis bag (Sigma-Aldrich Inc.) of a molecular weight cut-off 14 kDa. The dialysis bag was immersed in a glass bottle containing 400 mL of pH 7.4. The bottles were kept in a shaking water bath (Model 1031; GFL Corporation, Burgwedel, Germany) at 37 °C and 100 rpm. The parameters of the in vitro release study were selected to achieve the sink condition. Aliquots of 2 mL were taken from the receptor compartment at predetermined time intervals with immediate replacement. The quantity of RSV in the withdrawn samples was determined spectrophotometrically at 242 nm. The experiment was done in triplicate for each formulation and mean values were calculated.

### Nanoparticles flexibility

To evaluate the flexibility of the prepared NPs, the extrusion method was used [3]. Concisely, the optimized chitosomes nano-formulation and the corresponding liposomal NPs were separately extruded through 0.1 mm pore size membrane filter under reduced pressure. The size of both NPs was measured before and after the extrusion process. The flexibility was estimated as the percent change in the NPs size according to the following equation:

$$ Flexibility=\frac{Size\ of\ the\  NPs\  before\ extrusion- Size\ of\ the\  NPs\  after\ extrusion}{Size\ of\ the\  NPs\  before\ extrusion}\times 100 $$

### Cell viability and cytotoxicity assay

This test aimed to determine the effect of the prepared RSV lipid-based NPs formulations on the intestinal cell viability and cytotoxicity. The potential effects of both formulations on the human colorectal (HCT-116) cells were assessed by 3-(4,5-dimethylthiazol-2-yl)2,5 -diphenyl tetrazolium bromide (MTT) assay. Cells were seeded on 96-well plates (1 × 10^4^ cells/well) and incubated at 37 °C under a humidified atmosphere of 5% CO_2_ for 24 h. The cell medium was then changed to serum free medium (SFM) alone or SFM containing RVS loaded liposomal NPs and chitosomes nano-formulation in different drug concentrations (1.95, 3.91, 7.81, 15.63, 31.21, 62.5, 125 and 250 μM) and incubated for 72 h at 37 °C. Each formulation was compared with a drug-free carrier as a negative control. After incubation, SFM in the control and test wells were replaced by 100 μL/well of MTT solution (0.5 mg/ml) in PBS and incubated at 37 °C for another 3 h. The MTT solution was removed and the purple formazan crystals formed at the bottom of the wells were dissolved using 100 μL DMSO/well with shaking for 2 h at room temperature. The intensity of the color obtained was measured at 549 nm using a microplate reader (ELX 800; Bio-Tek Instruments, Winooski, VT, USA). Data, expressed as the percentages of viable cells, were compared to the survival of a control group. Values of the half maximal inhibitory concentration (IC_50_) were also estimated. Cells treated with DMSO only were defined as 100%.

## Results

A great number of pharmacologically active compounds do not reach the commercialization step simply because of their limited oral bioavailability that is attributed to inadequate dissolution rate. A substantial problem that is currently confronting the pharmaceutical industry for drugs of limited aqueous solubility is mainly attributed to their limited dissolution rate [7]. Also, during the first-pass metabolism phenomenon, the fraction of drug administered is lost during the absorption process due to the liver and/or gut wall metabolism. Accordingly, the drug concentration is greatly reduced before reaching the systemic circulation [35].

Chitosan-based NPs have been successfully developed and reported to have wide applications especially in oral drug delivery [15, 19, 28, 41]. The reported benefits include; enhancing transport of active pharmaceutical ingredients across the intestinal epithelial cell layer, protection of insulin against degradation in the gastrointestinal fluid, enhancement of bioavailability and improvement of aqueous drug solubility. In this study, flexible liposomal NPs and their corresponding chitosomes nano-formulation loaded with RSV were prepared to develop new nanocarrier systems suitable to investigate their role in intestinal cells cytotoxicity and improving RSV bioavailability.

### Characterization of the flexible lipid-based NPs formulations

Flexible liposomal NPs were prepared using the lipid film hydration technique utilizing L-α phosphatidylcholine, dicetyl phosphate and tween 80 as the main component of the liposomes membrane, negative charge inducing agent and edge activator, respectively. These flexible liposomal NPs were subsequently coated with chitosan to produce chitosan-coated flexible liposomes that have been assigned the name chitosomes nano-formulation. Both NPs formulations have been characterized for size, PDI, EE and zeta potential. Results for these parameters are illustrated in Table 1. The diameter size of the obtained chitosomes nano-formulation was in the range of 112.19 ± 2.90–554.33 ± 11.06 nm, the PDI was between 0.258 ± 0.023–0.581 ± 0.051, the EE was ranged between 84.78 ± 3.09–94.59 ± 1.62% and the obtained zeta potential value, which indicates surface charge of the particles, was in the range of (− 10.07 ± 1.26)- (+ 28.87 ± 1.39) mV. Characterization of the corresponding flexible liposomal NPs revealed a vesicle size between 96.73 ± 2.45–282.33 ± 10.22 nm, PDI values in the range 0.3213 ± 0.0290–0.5423 ± 0.0236, an EE between 71.44 ± 2.48–79.57 ± 1.27% and negative zeta potential values of (− 8.84 ± 0.66)- (− 12.73 ± 0.81).

### Experimental design statistical analysis results

Statistical analysis for the effect of the independent factors (X_1_, X_2_ and X_3_) on the dependant responses (Y_1_, Y_2_ and Y_3_) was accomplished by multiple regression analysis and two- way analysis of variance (ANOVA) using the StatGraphics software. Regression analysis is a statistical processes that estimate and analyze the relationships between a dependent variable and one or more of the independent variables. The two-way ANOVA aims to assess the main, interaction and quadratic effect of the independent variable on one dependent response. Accordingly, estimated effect of factors, F-ratios, and associated *P*-values were calculated and the obtained data are presented in Table 2. The sign of the estimated effect is an indication of a synergistic (positive sign) or antagonistic (negative sign) effect of this factor on the studied response. F-ratio is used to compare between the observed and expected averages. An F-ratio greater than 1 specifies a location effect and hence the *P*-value is used to report the significant level. If the obtained results for the *P*-value, for an independent factor, differs from zero and is less than 0.05, this factor is significantly affecting the studied response.

**Table 2:** Estimated effects of factors, F-ratios, and associated *P*-values for the studied responses Y_1_-Y_3_

Factor	Y_1_			Y_2_			Y_3_		
	Estimated effect	F-ratio	*P*-Value	Estimated effect	F-ratio	*P*-Value	Estimated effect	F-ratio	*P*-Value
X_1_	21.415	6.68	0.0492^a^	−1.67	6.08	0.0569	1.568	0.75	0.4262
X_2_	−45.985	30.80	0.0026^a^	−1.825	7.26	0.0431^a^	1.638	0.82	0.4073
X_3_	714.135	2252.15	0.00001^a^	14.160	132.45	0.0001^a^	35.187	114.48	0.0001^a^
X_1_X_1_	−8.209	0.68	0.4472	3.333	16.76	0.0094^a^	0.067	0.00	0.9767
X_1_X_2_	−25.09	6.88	0.0470^a^	−0.78	0.99	0.3645	3.455	2.73	0.1593
X_1_X_3_	7.375	0.38	0.5644	1.5	2.35	0.1856	−0.944	0.13	0.7328
X_2_X_2_	−13.884	1.94	0.2220	3.953	23.58	0.0047^a^	1.932	0.79	0.4154
X_2_X_3_	2.506	0.04	0.8423	0.113	0.01	0.9129	−4.363	2.79	0.1559
X_3_X_3_	266.368	293.08	0.00001^a^	7.115	31.27	0.0025^a^	−9.092	7.15	0.0442^a^
R^2^ (%)	99.87			97.99			99.09		
Adj. R^2^ (%)	99.64			94.38			97.47		
SE of Est.	9.57			0.782			2.091		
Mean AE	4.82			0.406			1.058		

**Abbreviations:** *X*_*1*_ Drug to phospholipid, *X*_*2*_ Surfactant concentration, *X*_*3*_ Coating solution concentration, *Y*_*1*_ Particle size (nm), *Y*_*2*_ Entrapment efficiency (%), *Y*_*3*_ Zeta potential (mV), *X*_*1*_*X*_*2*_*, X*_*1*_*X*_*3*_*, and X*_*2*_*X*_*3*_ are the interaction effects of the studied factors, *X*_*1*_*X*_*1,*_ *X*_*2*_*X*_*2*_ *and X*_*3*_*X*_*3*_ are the quadratic effects of factors, *SE* Standard error, *AE* Absolute error

**Note:** ^a^ indicates significant effect of this factor on the studied response

#### Effect of the drug to phospholipid molar ratio (X_1_) on the studied responses (Y_1_-Y_3_)

**Fig. 1:**
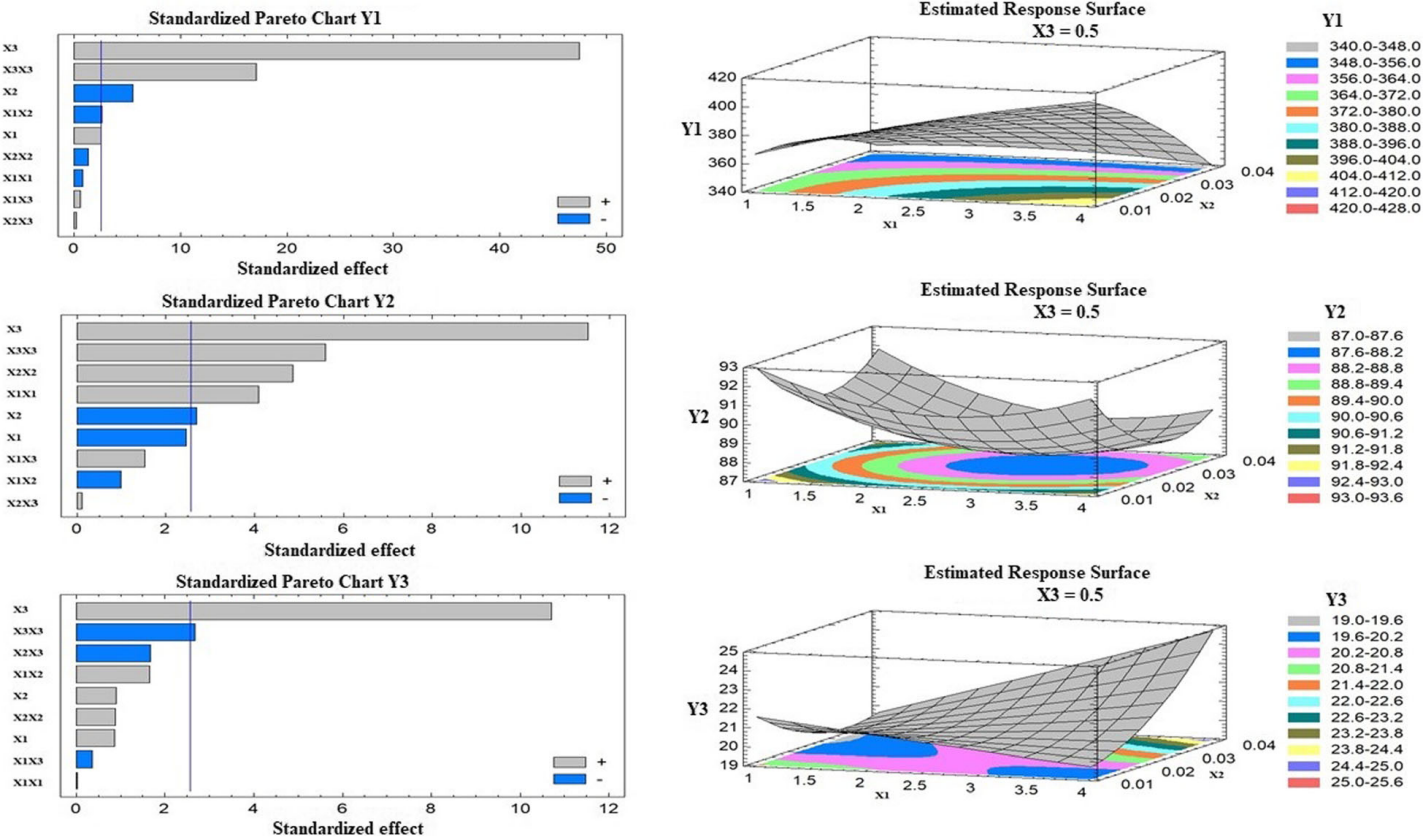
Standardized Pareto charts and response surface plots for the effect of the studied factors on Y_1_-Y_3_. ***Abbreviations:*** X_1,_ Drug to phospholipid; X_2,_ Surfactant concentration; X_3,_ Coating solution concentration; Y_1,_ Particle size (nm); Y_2,_ Entrapment efficiency (%); Y_3,_ Zeta potential (mV); X_1_X_2_, X_1_X_3_, and X_2_X_3_ are the interaction effects of the studied factors; X_1_X_1,_ X_2_X_2_ and X_3_X_3_ are the quadratic effects of factors

Results of the statistical analysis illustrated in Table 2, indicated that the drug to phospholipid molar ratio (X_1_) was significantly affecting the particle size (Y_1,_) at *P*-values of 0.0492. The pareto chart, displayed in Fig. 1, also confirmed this finding. A reference line is displayed in this chart. Any bar that extends after this line confirms the significant effect of this factor on the studied response. As indicated from the sign of the estimated effect and the pareto chart, X_1_ exhibited an agonistic effect on Y_1_.

#### Effect of the surfactant concentration (X_2_) on the studied responses (Y_1_-Y_3_)

The surfactant concentration (X_2_) was significantly affecting the particle size (Y_1,_) and the EE (Y_2_) at *P*-values of 0.0026 and 0.0431, respectively as indicated by the pareto (Fig. 1) and the values of the estimated factors effects. X_2_ was antagonistically affecting Y_1_ and Y_2_. Hence, increasing the surfactant concentration decreased the particle size and the EE of the prepared NPs.

#### Effect of the chitosan solution concentration (X_3_) on the studied responses (Y_1_-Y_3_)

It was noticed that the concentration of the chitosan solution (X_3_) was significantly affecting all the studied response (Y_1_-Y_3_). This factor illustrated a synergistic, positive, effect on Y_1_, Y_2_ and Y_3_. Bars in the pareto chart, Fig. 1, confirmed this finding.

It was noticed from the ANOVA that the interaction effect of X_3_X_3_ significantly affected all the studied variables. X_1_X_1_, and X_2_X_2_ were significantly affecting Y_2_, while X_1_X_2_ was affecting Y_1_.

The mathematical model for the studied responses was generated and the polynomial equations that best fit the models are:

$$ {\mathrm{Y}}_1=167.497+25.282\ {\mathrm{X}}_1+1236.66\ {\mathrm{X}}_2-735.615\ {\mathrm{X}}_3-1.82426\ {{\mathrm{X}}_1}^2-557.556\ {\mathrm{X}}_1{\mathrm{X}}_2+9.83333\ {\mathrm{X}}_1{\mathrm{X}}_3-30853.7\ {{\mathrm{X}}_2}^2+334.167\ {\mathrm{X}}_2{\mathrm{X}}_3+2130.95\ {{\mathrm{X}}_3}^2. $$

$$ {\mathrm{Y}}_2=102.625-4.8270{4\mathrm{X}}_1-464.259\ {\mathrm{X}}_2-33.9708\ {\mathrm{X}}_3+0.740741\ {{\mathrm{X}}_1}^2-17.3333\ {\mathrm{X}}_1{\mathrm{X}}_2+2.0\ {\mathrm{X}}_1{\mathrm{X}}_3+8785.19\ {{\mathrm{X}}_2}^2+15.0\ {\mathrm{X}}_2{\mathrm{X}}_3+56.9167\ {{\mathrm{X}}_3}^2. $$

$$ {\mathrm{Y}}_3=-36.8913-0.841852\ {\mathrm{X}}_1-61.1574\ {\mathrm{X}}_2+160.79\ {\mathrm{X}}_3+0.0148148\ {{\mathrm{X}}_1}^2+76.7778\ {\mathrm{X}}_1{\mathrm{X}}_2-1.25833\ {\mathrm{X}}_1{\mathrm{X}}_3+4292.59\ {{\mathrm{X}}_2}^2-581.667\ {\mathrm{X}}_2{\mathrm{X}}_3-72.7292\ {{\mathrm{X}}_3}^2. $$

Figure 1 also illustrates the three-dimensional estimated response surface plots that demonstrate the effect of two independent factors on a studied response when the value of the third factor was kept at intermediate level. Individual analysis for the effect of the studied variables on the Y_1_ indicated that to prepare flexible chitosomes nano-formulation of 99.12 nm, the optimum levels for X_1_, X_2_ and X_3_ should be 1.0, 0.04 and 0.2, respectively as illustrated in Table 3. To obtain vesicles of 95.99% EE, the optimum levels for X_1_, X_2_ and X_3_ should be 1.0, 0.01 and 0.6, respectively. Flexible chitosomes nano-formulation with a zeta potential value of 28.85 mV could be obtained at 1.0, 0.01 and 0.59 of X_1_, X_2_ and X_3_, respectively. After analyzing the multiple effect of the studied variables on Y_1_, Y_2_ and Y_3_, it was assumed that the optimum desirability that achieve the smallest particle size, highest EE and highest zeta potential values could be achieved at X_1_, X_2_ and X_3_ levels of 1.0, 0.01 and 0.47, respectively. An optimized chitosomes nano-formulation formulation that contains these values was prepared, characterized and the values for predicted and observed values and their residuals are depicted in Table 3**.** The corresponding flexible liposomal NPs showed an average size of 230.34 ± 8.73 nm, PDI value of 0.508 ± 0.075, zeta potential value of − 10.29 ± 0.46 mV and EE of 78.13 ± 0.54%.

**Table 3:** The optimum levels and values, and desirability levels and values for the studied factors and responses

Factors	Low	High	Optimum level for each response			Optimum desirability level
			Y_1_ = 99.12 nm	Y_2_ = 95.99%	Y_3_ = 28.85 mV	
**X**_**1**_ **(MR)**	1.0	4.0	1.0	1.0	1.0	1.0
**X**_**2**_ **(%)**	0.01	0.04	0.04	0.01	0.01	0.01
**X**_**3**_ **(%)**	0.2	0.6	0.2	0.6	0.59	0.47
**Responses**	**Goal**		**Optimum desirability**			
			**Predicted values**	**Observed values**		**Residual**
**Y**_**1**_ **(nm)**	Minimize		331.09	342.33		11.24
**Y**_**2**_ **(%)**	Maximize		92.31	94.01		1.7
**Y**_**3**_ **(mV)**	Maximize		19.39	21.22		1.83

**Abbreviations:** *X*_*1*_ Drug to phospholipid, *X*_*2*_ Surfactant concentration, *X*_*3*_ Coating solution concentration, *Y*_*1*_ Particle size, *Y*_*2*_ Entrapment efficiency, *Y*_*3*_ Zeta potential, *MR* Molar ratio

### Morphological characterization

**Fig. 2:**
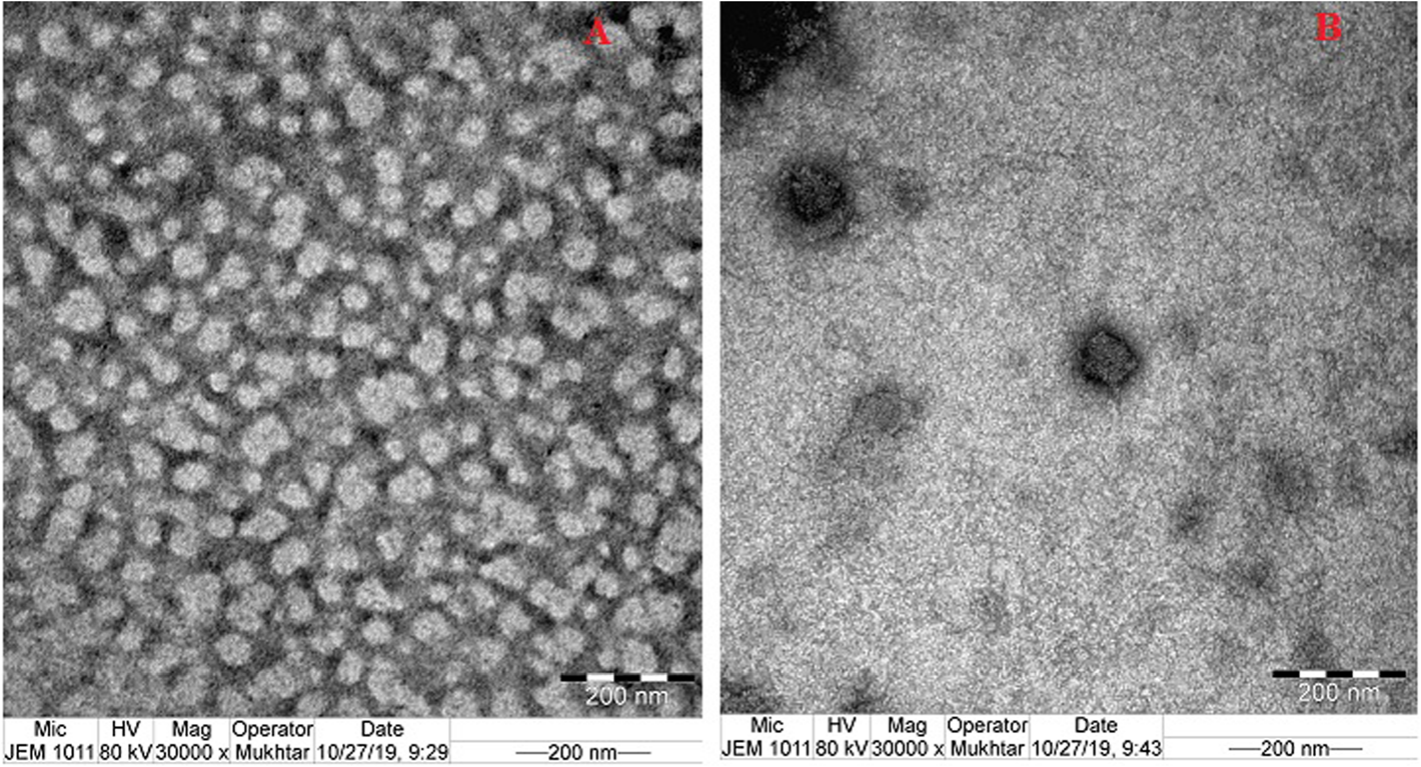
TEM images of rosuvastatin liposomal NPs (**a**) and chitosomes nano-formulation (**b**)

The vesicular nature of the liposomal NPs and chitosomes nano-formulation was confirmed after investigation of their morphological characteristics as depicted in Fig. 2. Both formulations showed spherical shape particles with a marked outer shell for chitosomes nano-formulation. Addition of chitosan during preparation of the lipid-based vesicles resulted in deposition of this cationic polymer on the particles outer surface, the effect that lead to formation of thick outer membrane. Moreover, chitosomes nano-formulation exhibited larger size compared to the corresponding liposomal NPs which is in a good agreement with the results obtained from the particle size analysis.

### Fourier transforms infrared (FT-IR)

In this work, we have studied the FT-IR spectrum for RSV loaded into different lipid-based nano-dispersed formulation without drying. We aimed to identify the drug physicochemical changes in the developed liposomal NPs and chitosomes nano-formulation without any further modifications in the formulation nature.

**Fig. 3:**
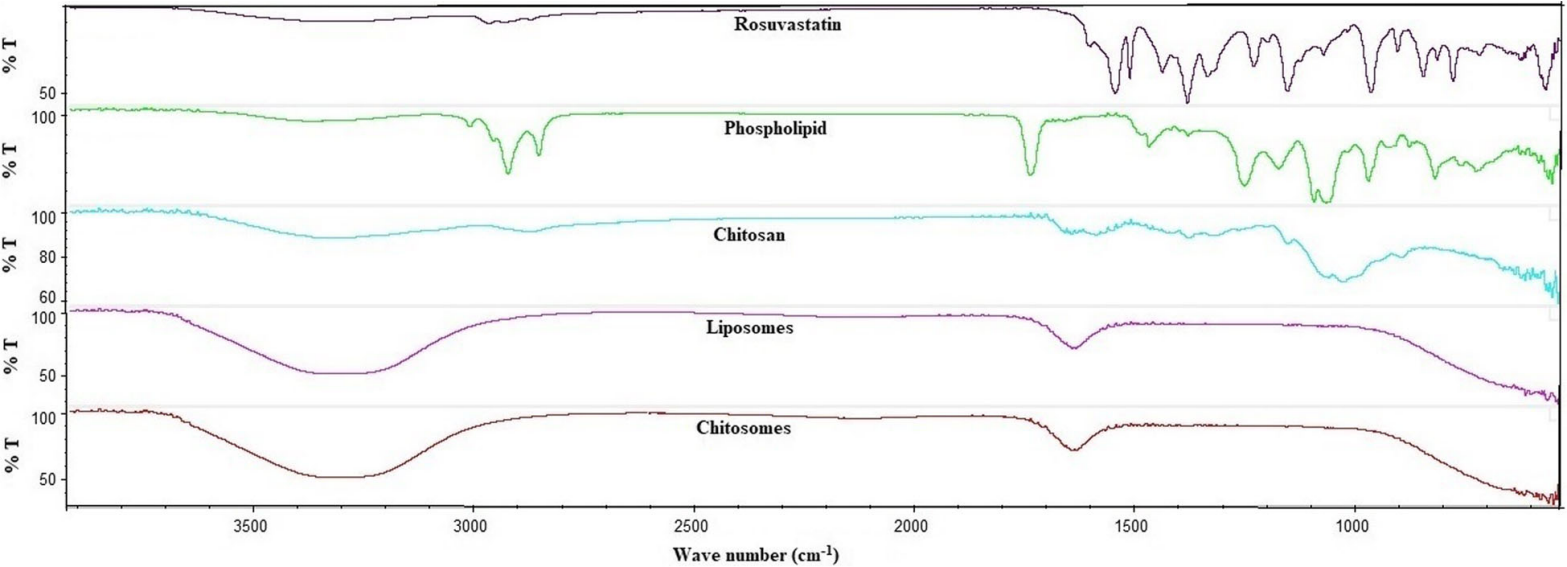
Fourier-transformed infrared spectra of rosuvastatin, phospholipid, chitosan, liposomal NPs and chitosomes nano-formulation

The FT-IR spectrum of pure RSV (Fig. 3) illustrated a broad band for O-H stretching at 3380 cm^− 1^ and a band at 2920 cm^− 1^ for = C-H stretching. Another two drug peaks were detected at 1550 cm^− 1^ and 1515 cm^− 1^ corresponding to C=C stretching and N-H bending, respectively. Asymmetric and symmetric bending vibration of the drug CH_3_ group were noticed at 1485 cm^− 1^ and 1380 cm^− 1^, respectively. The asymmetric vibration of S=O was observed at 1330 cm ^1^. The bending vibrations for C-H and C-F stretching were identified at 1230 cm^− 1^ and 1155 cm^− 1^, respectively.

The spectrum of the L-α phosphatidylcholine illustrated a broad peak at 3354 cm^− 1^ corresponding to the stretching vibration of the hydroxyl (OH) group. A sharp characteristic band between 2700 and 2950 cm^− 1^ was originated from the C-H stretching vibration of the aliphatic methyl group. Another peak at 1735 cm^− 1^ that assigned to C=O stretching was also detected. The PO_2_ antisymmetric double bond stretching bands was noticed at 1250 cm^− 1^. Chitosan showed a broad band in the range of 3300–2900 cm^− 1^ corresponded to the amine and hydroxyl groups. A peak at 2876 cm^− 1^ that is attributed to -OH stretching; a characteristic band of the carbonyl (C=O) stretching of the secondary amide was detected at 1655 cm^− 1^, and the bending vibrations of the N-H (N-acetylated residues, amide II band) was observed at 1599 cm^− 1^ [38] [38] [38] [38] [38] [38] [38]. Other peaks at 1423 and 1381 cm^− 1^ that belong to the N-H stretching of the amide and ether, respectively were also detected.

The spectrum of the liposomal NPs and chitosomes nano-formulation confirmed the physical interaction between the studied components and effective encapsulation of RSV in the prepared nanovesicles. Broadening, overlapping, weak and/or disappearance of some bands in the spectra of the NPs formulation were noticed. These changes were observed for phospholipid bands between 2700 and 2950 cm^− 1^, and at 1735 cm^− 1^; for drug peaks at 1515 and 1330 cm ^1^; for chitosan peaks at 1599, 1423 and 1381 cm^− 1^. Phosphatidyl choline is a neutral or zwitterionic substance over a wide pH range from strongly acid to strongly alkaline.

### In vitro release study

**Fig. 4:**
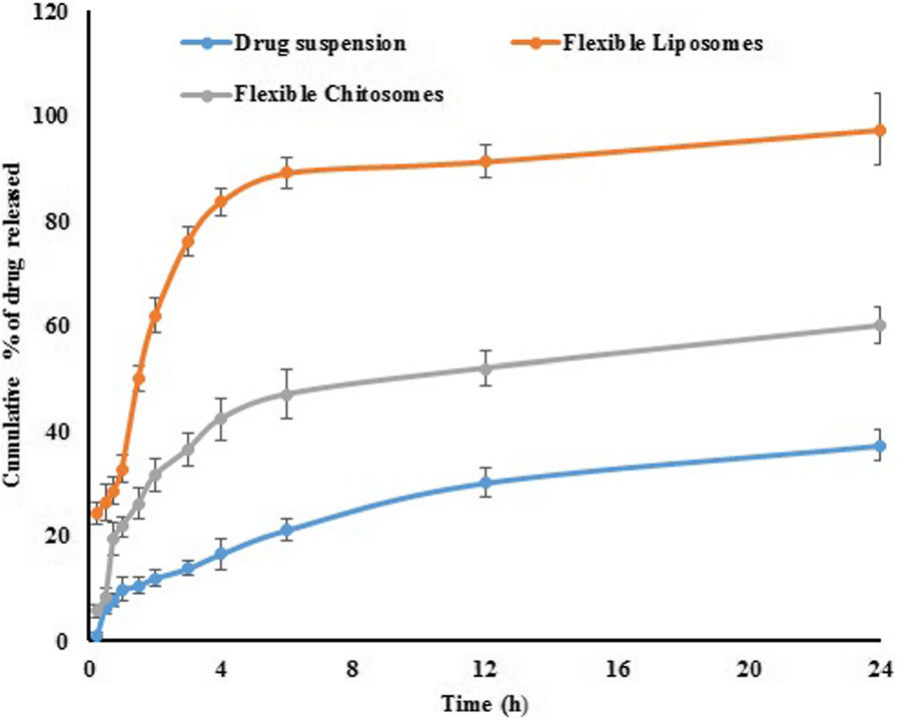
In vitro release of rosuvastatin from pure drug suspension, liposomal NPs and chitosomes nano-formulation

The in vitro release of RSV from the prepared liposomal NPs and chitosomes nano-formulation was studied and compared to that of a pure drug suspension using the dialysis bag technique. As depicted in Fig. 4, the release of RSV from the liposomal NPs illustrated a biphasic drug release pattern. An initial rapid drug release rate that was followed by a slow drug release pattern. The optimized flexible chitosomes nano-formulation exhibited a lower initial drug release, compared to the corresponding liposomal NPs, the effect that is explained by the high EE (chitosomes nano-formulation EE = 94.01%). A second sustained release stage was observed for both liposomal NPs and chitosomes nano-formulation. Drug suspension demonstrated an overall low drug release pattern, when compared to lipid-based NPs formulations, and smaller cumulative drug release due to the presence of the suspended drug particles in a coarse dispersion state.

### NPs flexibility

The ability of the prepared nanovesicles to pass through 0.1 mm pore size membrane filter under reduced pressure was considered as a measurement of NPs flexibility. The prepared liposomal NPs and chitosomes nano-formulation revealed a flexibility value of 44.43 and 40.16%, respectively.

### Cell viability and cytotoxicity assay

Statins possess anticancer activity, against many cancer cells, under in vitro conditions in a time- and dose-dependent manner [29]. Previous studies mentioned that RSV demonstrated a cytotoxic activity against thyroid, hepatic, breast, cervical and prostate cancer cell lines [10, 44]. Another study illustrated the anti-melanoma properties of RSV [29]. The anticancer activity of statins is attributed to their ability to inhibit the mevalonate pathway, which leads to reduction of cholesterol synthesis, and to their ability to decrease the cellular levels of non-steroidal isoprenoids, geranylgeranyl pyrophosphate and farnesyl pyrophosphate. These effects result in failure of protein prenylation which affects carcinogenesis [29]. As far as we know, RSV activity against intestinal cells has not been studied to date.

**Fig. 5:**
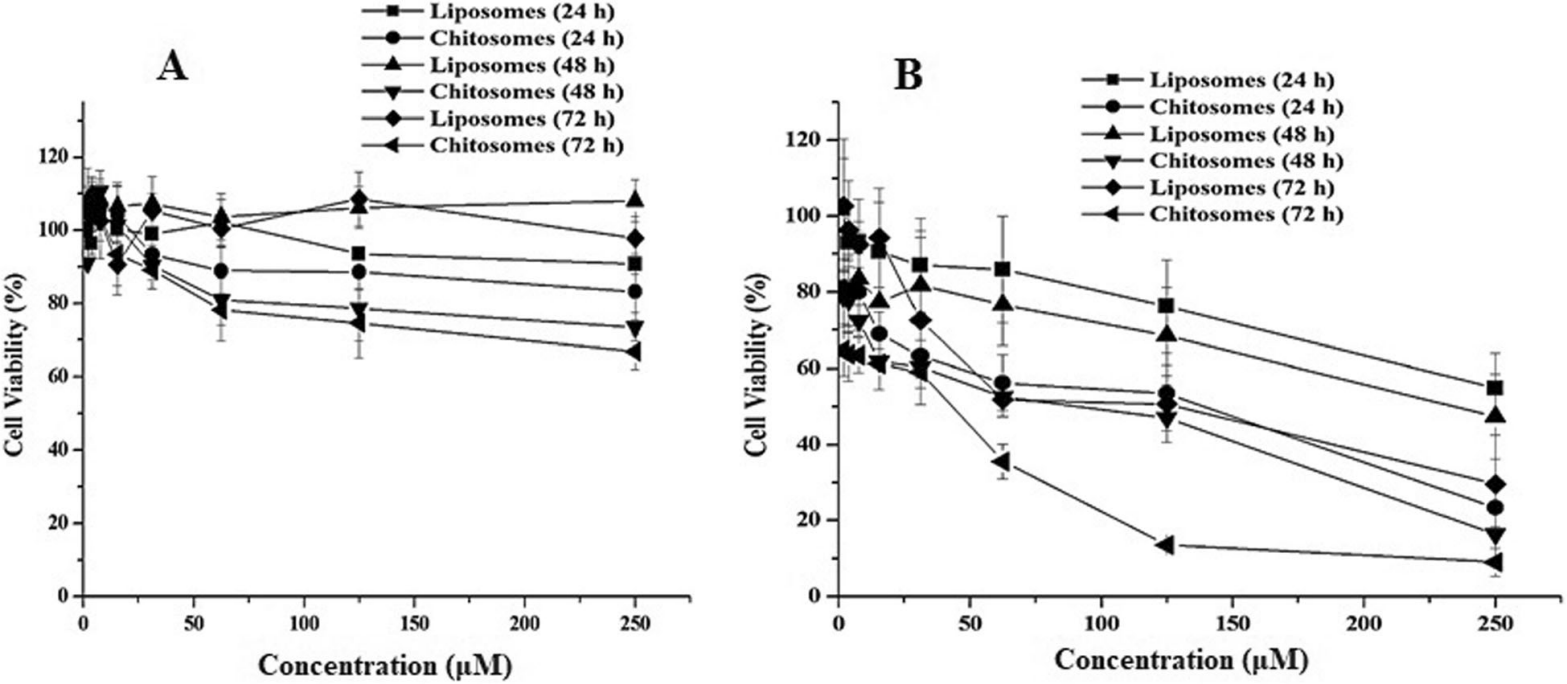
Cell viability after treatment with free liposomal NPs and chitosomes nano-formulation (**a**), and following treatment with rosuvastatin loaded liposomal NPs and chitosomes nano-formulation (**b**)

To examine the biocompatibility and cytotoxic effects of plain and drug loaded liposomal NPs or chitosomes nano-formulation, HCT-116 cells were treated with different concentrations of these samples for 3 days and the MTT assay was performed to investigate the effect of these formulation on the viability of living cells. This test depends on the production of a colored formazan by the action of the viable cells’ mitochondrial enzymes on MTT. Cells were exposed to increasing RSV concentrations of drug loaded liposomal NPs and chitosomes nano-formulation standardized at certain RSV concentrations. Cells were also incubated with blank liposomal NPs and chitosomes nano-formulation to exclude the effect of RSV. As shown in Fig. 5, cellular proliferation was inhibited in a dose-dependent manner of RVS. The inhibition difference between plain (drug free) chitosomes and liposomes treated with 250 μM at 24 h was much less than its drug-loaded counterpart, the effect that could be attributed to the prominent drug cytotoxic effect at this concentration.

Interestingly, the IC50 values of chitosomes nano-formulation were found to be smaller than the corresponding liposomal NPs. The calculated IC50 values for chitosomes nano-formulation were about 142.5, 98.6 and 43.9 μM after 24, 48 and 72 h respectively, while that of liposomal NPs were 234.8, 218.8 and 131.7 μM respectively, after the same.

## Discussion

As the drug to phospholipid molar ratio was increased from 1: 1 to 1: 4, the particle size of the prepared NPs increased. At higher phospholipid load, the formation of multilamellar vesicles is favored, the effect that results in increasing the size of the prepared NPs. This explanation was previously mentioned by Harbi et al. during development of sertraline liposomes [23].

The effect of the surfactant concentration on the particle size could be attributed to the reduction of the surface tension of the media at higher surfactant concentration and so arrangement of the phospholipid in small vesicles as previously reported [5]. The decrease in the EE of the prepared vesicles that was observed at higher surfactant concentration could be attributed to the formation of micelles molecules that compete for the drug molecules with the lipid vesicles. This finding is in a good agreement with the work done by Patel et al., who reported a decrease in the EE of curcumin loaded transfersomes with increasing the surfactant concentration [33].

The particle size, EE and zeta potential of the drug loaded NPs were increased as the concentration of chitosan (coating) solution was increased. Addition of chitosan to the flexible liposomal vesicles resulted in coating of the NPs outer surface by an electrostatic interaction between the positively charged chitosan and the negatively charged NPs surface. This coating process resulted in an enlargement of the vesicles as previously stated [32, 46]. Deposition of more chitosan on the NPs surface was achieved at higher polymeric concentration, the effect that results in increasing the zeta potential value and promotion of more drug entrapment in the coated NPs. The electrostatic interaction between the negatively charged drug and the positively charged chitosan, that deposited on the NPs surface, may be another cause for the increase in drug EE at higher chitosan concentration.

Encapsulation of RSV in the phospholipid molecules, adsorption of the DCP at the phospholipid molecules and chitosan coating of the flexible liposomal NPs may result in electrostatic interaction by weak van der Waals force of attraction or dipole–dipole and hydrogen bond formation. This finding was confirmed after FTIR characterization and was previously reported by Rudra et al., during development and characterization of nanoliposomes loaded with doxorubicin [36].

The amount of drug that was released from the liposomal NPs during the early stage is mainly attributed to the un-encapsulated drug (liposomal NPs EE = 78.13 ± 0.54%), drug adsorbed on the NPs surface and to the drug permeated from the prepared NPs. Chitosomes nano-formulation illustrated an extended drug release pattern due to the presence of chitosan in the outer shell which delayed the diffusion of RSV into the release medium. This effect was previously reported by Chen et al., who illustrated the role of chitosan coating on the behavior of flurbiprofen-loaded chitosan-coated deformable liposomes [16]. The second sustained release stage that was observed for both liposomal NPs and chitosomes nano-formulation could be attributed to encapsulation of RSV within the lipid shell that allows slow drug release from the lipid matrix. This finding is in a good agreement with that reported by Perez et al., during development of amphotericin B ultra-deformable liposomes [34]. After 24 h, the values for drug release from liposomal NPs, chitosomes nano-formulation and pure drug suspension were 97.54 ± 3.37, 59.98 ± 3.47 and 37.28 ± 2.86%, respectively.

The flexibility values of both formulations indicated the elasticity of the prepared NPs. The effect that is attributed to the presence of the edge activator component. The presence of the edge activator weakens the phospholipid bilayer and render the nano-vesicle ultra-deformable. Chitosomes nano-formulation demonstrated less flexibility than the corresponding liposomal NPs, the effect that is attributed to the chitosan coating process.

The cellular proliferation was inhibited in a dose-dependent manner of RVS which is an indication for the reduction in cell viability upon increasing the drug concentration in the prepared liposomal NPs and chitosomes nano-formulation. This finding is in a good agreement with earlier studies for other statins such as lovastatin [40] and simvastatin [37]. The smaller IC50 values of chitosomes nano-formulation when compared to the corresponding liposomal NPs indicate a gradual release of RSV from the NPs phospholipid bilayers and the significant effect of chitosan layer on cell viability when compared to liposomal NPs. Positivity of the NPs outer shell demonstrated a marked cytotoxic effect which was also obvious with plain (drug free) chitosomes nano-formulation but to a lesser extent. This finding indicates the higher therapeutic window of RSV chitosomes nano-formulation. Accordingly, chitosomes nano-formulation could be considered as a good nanocarrier for RSV cytotoxic activity while drug loaded liposomal NPs could be considered as a suitable carrier to enhance the activity of RSV in treatment of cardiovascular diseases, but more investigations are required.

## Conclusions

The optimization design was successfully used to develop RSV lipid-based nanocarriers of spherical shape. Chitosomes nano-formulation illustrated a distinct outer shell membrane and larger size vesicles when compared to the corresponding liposomal NPs. Both formulations illustrated a biphasic drug release profile. Chitosomes nano-formulation exhibited a lower initial and more extended drug release pattern when compared to liposomal NPs. Chitosomes nano-formulation demonstrated smaller IC50 values than the corresponding liposomal NPs. Both NPs formulations exhibited an anticancer activity in a time- and dose-dependent manner. Chitosomes nano-formulation are a promising nanocarrier for RSV cytotoxicity while liposomal NPs are appropriate carrier to enhance RSV bioavailability in treatment of hyperlipidemia.

## Data Availability

All data obtained and analyzed during this work are included in this article. Extra information is available upon reasonable request from the corresponding author.

## Abbreviations

ANOVA: Analysis of variance
DCP: Dicetyl phosphate
EE: Entrapment efficiency
FT-IR: Fourier transforms infrared
HMG-CoA: 3-hydroxy-3-methyl-glutaryl-CoA
IC50: The half maximum inhibitory concentrations
LDL: Low-density lipoprotein
MTT: 3-(4,5-dimethylthiazol-2-yl)2,5 -diphenyl tetrazolium bromide
NPs: Nanoparticles
PDI: Polydispersity index
RSV: Rosuvastatin
TEM: Transmission electron microscopy
X_1_: Drug to phospholipid
X_2_: Surfactant concentration
X_3_: Coating solution concentration
Y_1_: Particle size (nm)
Y_2_: Entrapment efficiency (%)
Y_3_: Zeta potential (mV); X_1_X_2_, X_1_X_3_, and X_2_X_3_ are the interaction effects of the studied factors; X_1_X_1,_ X_2_X_2_ and X_3_X_3_ are the quadratic effects of factors
